# Central airway obstruction treatment with self‐expanding covered Y‐carina nitinol stents: A single center retrospective analysis

**DOI:** 10.1111/1759-7714.14359

**Published:** 2022-02-24

**Authors:** Arik Bernard Schulze, Georg Evers, Friederike Sophia Tenk, Christoph Schliemann, Lars Henning Schmidt, Dennis Görlich, Michael Mohr

**Affiliations:** ^1^ Department of Medicine A Hematology, Oncology and Pulmonary Medicine, University Hospital Muenster Muenster Germany; ^2^ Medical Department IV Pulmonary Medicine and Thoracic Oncology, Klinikum Ingolstadt Ingolstadt Germany; ^3^ Department of Internal Medicine II University Hospital Regensburg Regensburg Germany; ^4^ Institute of Biostatistics and Clinical Research Westfaelische‐Wilhelms University Muenster Muenster Germany

**Keywords:** bronchial stent, CAO, nitinol Y‐carina stent, self‐expanding stent

## Abstract

**Background:**

Central airway obstruction (CAO) is one of the most challenging, potentially lethal complications in malignant and benign respiratory diseases. Worsening dyspnea is also a relevant cause for reduced quality of life in such patients. Here, we present our data on the application of covered, self‐expanding Y‐carina nitinol stents due to benign and malignant diseases.

**Methods:**

We retrospectively identified 27 patients who had undergone 31 rigid bronchoscopies with implantation of covered Y‐carina nitinol stents over a period of 10 years in order to evaluate indication, clinical course, and outcome.

**Results:**

Short‐term survival of successfully stented patients with palliative and curative treatment goal did not differ, allowing for diagnosis independent indication. With respect to overall survival, patients with endoluminal obstruction benefited most compared to patients with fistula and/or external compression. Granulation tissue formation (61.3%) and mucus plugging (80.6%) were the most frequent complications. Material defect (6.5%) and migration (3.2%) were rare complications that could be handled by revisional rigid bronchoscopy and stent exchange in some cases.

**Conclusions:**

Implantation of self‐expanding covered Y‐carina nitinol stents via rigid bronchoscopy is a feasible and safe treatment option for benign and malignant central airway obstruction. Especially in palliative, malignant airway stenosis, stenting might facilitate additional treatment options and optimize dyspnea and eventually quality of life.

## Graphical abstract

Implantation of self‐expanding covered Y‐carina nitinol stents via rigid bronchoscopy in central airway obstruction is a feasible and safe treatment option for benign and malignant central airway obstruction, that might facilitate subsequent additional therapeutic options. Underlying diagnosis did not alter the outcome. Hence, indication as well as stent model selection should be discussed interdisciplinary with respect to expectable complications, benefits, and individual aims.

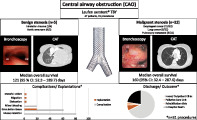

## INTRODUCTION

Progressive central airway obstruction (CAO) often results in life threatening circumstances. Patients may present with aggravating dyspnea^1^ and objectifiable reduced oxygen saturation,^2^ possibly amounting to life threatening respiratory failure.^3^ Especially in malignant causes, comorbid fistulas might also cause acute hemoptysis,^1^ pneumothorax or recurrent aspiration pneumonitis.^4^ Next to reduction of dyspnea and improvement in quality of life and physical capability,^1^ survival benefits are conceivable upon stent placement.^5^, ^6^ Moreover, stenting might allow for additional treatment options of the underlying disease. Hence, previously reported guidelines on lung cancer recommend using tracheobronchial stenting in cases of CAO as palliative treatment in combination with other treatment options.^7^

Malignant causes of CAO include non‐small cell lung cancer (NSCLC) as well as small cell lung cancer (SCLC).^3^, ^8^ Other tumors, such as thyroid cancer, thymoma, thymus carcinoma, mediastinal lymphoma, mediastinal germ cell cancer or metastases might promote external compression. Malignant pleural mesothelioma and esophageal carcinoma additionally tend to present an infiltrative or destructive growth with endobronchial tumor infiltration and/or fistula.^9^

While devastating prognosis in malignant CAO is comprehensible, benign causes of CAO are nonetheless harmful. These include iatrogenic and traumatic causes as well as infectious and noninfectious inflammatory diseases such as tuberculosis, scleroma, fungal or viral infections, sarcoidosis, relapsing polychondritis (RPC), granulomatosis with polyangiitis (GPA), and amyloidosis.^10^ Moreover, tracheobronchomalacia (TBM), hematoma, aortic aneurysm compression and dynamic airway collapse might also cause symptoms of central airway obstruction.^6^, ^9^, ^11^

In general, treatment indications should be discussed by a multidisciplinary team^10^ with regard to potential complications and risks. However, implantation is mostly performed by well‐trained interventional pulmonary specialists.^12^

Treatment options offered for CAO include uncovered and covered metal stents, as well as silicone stents.^10^ Other than these, biodegradable stents are available, but they are used infrequently and their indication is still debatable.^13^

Covered and uncovered metal stents are available as self‐expandable, fixed‐diameter or balloon‐expanded versions. They provide a reduced risk of stent migration due to immediate contact with the mucosa, especially in malignant stenosis. Implantation can be performed subsequent to flexible bronchoscopy but rigid bronchoscopy should still be available.^14^ However, due to higher migration rates in benign stenosis (i.e., one third of cases)^14^ the US‐american food and drug administration (FDA) has stated an application warning in this context.^12^ However, covered metal stents are the most frequently used tracheobronchial stents worldwide.^13^

Alternatively, silicone stents come as a folded or unfolded product but invariably require a precedent rigid bronchoscopy as well as a rigid passage of the stenosis. The selection of the stent must include expectable and actual bronchus diameters, measured beforehand via CAT scan. Hence, balloon dilation or endoluminal debulking via electrocautery, argon plasma coagulation or laser ablation is often needed.^15^ Silicone stenting has become more popular among interventional pulmonologists, especially in Europe.^13^

Here, we present our clinical data on the application of covered nickel‐titanium (nitinol) self‐expanding Y‐stents (Leufen aerstent TBY) and discuss their indication and complications.

## METHODS

### Patients

In order to evaluate the indication, clinical course, and outcome of patients with critical central airway stenosis, we retrospectively identified 27 patients who underwent 31 rigid bronchoscopies with implantation of self‐expanding, covered nitinol Y‐carina stents (aerstent TBY, Product Code LMPI0009, Leufen medical GmbH) between 2007 and 2017 at our tertiary care University Hospital.

After obtaining approval from the ethical committee in Münster (registration no. 2017‐556‐f‐S), we performed a database query on OPS‐codes 5‐319, 5‐314 and 5‐339 from the hospital information system. Patient records were scanned manually and gathered in order to evaluate the use of aerstent TBY Y‐carina self‐expanding nitinol stents. Age at implantation, gender, main diagnosis, malignant or benign cause of central airway obstruction, date of implantation, date of discharge after implantation, follow‐up flexible and/or rigid bronchoscopies, use of noninvasive ventilation as well as date of last contact (as censored) or death (as event) were compiled. For case presentation, conclusive bronchoscopic photographs and CAT scans were documented separately and anonymously. Data were subsequently anonymized and evaluated.

### Method of implantation

All patients presented with a current computed axial tomography (CAT) scan of the thorax, necessary to determine stent sizes by measuring the inner tracheal diameter and diameter of the main bronchi as well as the distance between the main carina and entry of upper lobe bronchi. If possible, before actual implantation, a bronchoscopy with a flexible 6 mm diameter bronchoscope was performed to evaluate the cause, localization, and grade of central airway stenosis as well as the possibly required interventional treatment options (i.e., balloon catheter bronchoplasty, forceps extraction, electrocautery, cryoablation, argon plasma coagulation (APC), and neodymium‐doped yttrium aluminum garned [Nd:YAG] laser ablation^4^) during implantation in an endoscopy suite with sedation and local anesthesia. The self‐expanding, covered nitinol Y‐carina stent model (aerstent TBY) used can be obtained with a tracheal diameter of 16, 18 or 20 mm and tracheal length of 40 or 50 mm. The right main bronchus diameter of the stent can also be obtained in 12 or 14 mm with a length of 15 or 20 mm, and the left main bronchus diameter of the stent can be between 12 or 14 mm with a standard length of 30 mm. The manufacturer’s will also provide different sizes upon special request.

Implantation of the self‐expanding, covered nitinol Y‐carina aerstent TBY was performed in an operating room under total intravenous general anesthesia (TIVA) and relaxation via rigid bronchoscopy and with the assistance of fluoroscopy. After general patient positioning and induction of TIVA and relaxation under preoxygenation and denitrogenation with 100% fraction of inhaled oxygen (FiO_2_), head positioning was optimized for insertion of a 10 or 12 mm inner diameter rigid bronchoscope. After intubation, patient ventilation was performed either via jet ventilation or via traditional ventilator modes with mouth packing^16^ via the rigid bronchoscope. Following intubation with a rigid bronchoscope we regularly performed a flexible bronchoscopy via the rigid scope. Here, with necessary attention to FiO_2_ levels in the tracheobronchial system, preimplantation interventional procedures, such as balloon catheter bronchoplasty, forceps extraction, cryoablation, argon plasma coagulation (APC) and neodymium‐doped yttrium aluminum garned (Nd:YAG) laser ablation^4^ were performed to optimize stent positioning afterwards.

Implantation followed the over the wire principle. A flexible bronchoscope was used to separately place two colored, radiopaque 0.035 in./0.89 mm diameter and 1800 mm long guide wires (aerstent GWA, product no. [black] 901‐35‐180, product no. [black/yellow] 912‐35‐180, Leufen medical GmbH) into the right and left main bronchus. Fluoroscopy was used to control positioning of the wires before proceeding. Afterwards, the 0.315 in./8 mm diameter and 600 mm long insertion system was threaded onto the guide wires and fluoroscopically placed into the main carina. Under intermittent fluoroscopic control, first the left bronchial section, then the right bronchial part and finally the tracheal section was expanded. Flexible bronchoscopy as well as fluoroscopy was used to control correct positioning and continuity of the airways. In particular, occlusion of the right upper lobe bronchus had to be excluded. Possible moderate bleeding complications were managed via oxymetazoline or diluted epinephrine/norepinephrine instillation.

After extraction of the rigid tube, patients were either manually ventilated via handheld face mask, laryngeal mask or endotracheal tube until sufficient vigilance and spontaneous breathing was achieved, depending on the level of relaxation and need for ventilatory positive airway pressure or driving pressure.

Then, 24–72 h after implantation, a flexible bronchoscopy was performed at the endoscopy suite or bedside on the intensive care unit to control stent positioning, exclude mucus airway obstruction and sample for microbial bearings. Patients were prompted to inhale hyperosmolar saline solutions (0.9% sodium chloride) as well as salbutamol and ipratropium bromide at least six to 10 times a day and flutter airway devices were subsequently used to support mucus clearance.

### Statistical analysis

Raw counts, frequencies, mean plus/minus standard deviation (±SD) and median with interquartile range (IQR = 25–75 percentile) or 95% confidence interval (95% CI) were used to describe the overall cohort and procedural cohort. Two‐fold associations between categorical variables were analyzed via Fisher's exact test or χ^2^‐test, if applicable. χ^2^‐test was also used to compare between three‐fold independent nominal samples. Associations between normally distributed, continuous variables were either tested using unpaired *t*‐test or one way ANOVA, depending on the count of the sample characteristics. Overall survival (OS) was defined as time (days) between stent implantation and death or censoring. Univariate survival analyses compared OS between groups using Log Rank tests. Kaplan Meier curves were used to visualize survival differences. Data collection was performed using Microsoft Office Professional Plus 2010 Excel (released 2010, Microsoft Corp.). Analyses were performed using IBM SPSS Statistics Version 27 (released 2020, IBM Corp.).

## RESULTS

Over a period of 10 years (i.e., 2007–2017) 27 patients successfully received 31 aerstent TBY Y‐carina nitinol stent implantations at our institution (Table 1). In this cohort, median overall survival was 160 (95% CI: 76.9–243.1) days and median follow‐up was 942 (95% CI: 548.0–1336.0) days. Mean age at first implantation was 55.3 years and 78% of the patients were male.

**TABLE 1:** Baseline characteristics of the cohort

	*n* of total patients (27)	% of total (100.0)
Age at first stent implantation		
Mean (± SD)	55.3 (± 15.7)	
Median (IQR)	55.0 (51.0–66.0)	
Gender		
Male	21	77.8
Female	6	22.2
Diagnosis		
NSCLC	7	25.9
SCLC	1	3.7
Esophageal carcinoma	11	40.7
Pulmonary metastasis	3	11.1
Aortic aneurysm	4	14.8
Inhalation trauma	1	3.7
Intention		
Cure	11	40.7
Palliation	16	59.3
Urgency		
Life threatening	7	25.9
Subacute	20	74.1
Indication		
Endoluminal obstruction	9	33.3
External compression	8	29.6
Fistula	10	37.0
Number of Y‐carina nitinol stent implantations		
Single intervention (1 stent)	23	85.2
Repeated interventions (2 stents)	4	14.8
Number of post interventional bronchoscopies		
*n*_bronch_ = 1–5	16	59.3
*n*_bronch_ = 6–10	3	11.1
*n*_bronch_ = 11–15	4	14.8
*n*_bronch_ > 15 (i.e., 21, 37, 44, and 47)		14.8
Overall survival (days)		
Median (95% CI)	160 (76.9–243.1)	
Follow‐up (days)		
Median (95% CI)	942 (548.0–1336.0)	

*Note*: Esophageal carcinoma including squamous cell carcinoma and adenocarcinoma of the esophagus. Abbreviations: SD, standard deviation, IQR, interquartile range; 95% CI, 95% confidence interval; NSCLC, non‐small cell lung cancer including squamous cell carcinoma and adenocarcinoma of the lung; SCLC, small cell lung cancer.

### Patient characteristics

Underlying diseases ranged from malignant causes such as esophageal cancer (*n* = 11, 41%), lung cancer (*n* = 8, 30%), and pulmonary metastasis (*n* = 3, 11%; i.e., clear cell renal cell carcinoma, osteosarcoma, and head and neck squamous cell carcinoma) to benign causes, including inhalation trauma (*n* = 1, 4%) and thoracic aortal aneurysm with compression atelectasis (*n* = 4, 15%). In 59% of the cases, stenting indication was a palliative treatment option. However, implantation in acute and life‐threatening situation was performed in seven cases (26%). Interventional cause was either due to tracheoesophageal fistula (*n* = 10, 37%), endoluminal obstruction (*n* = 9, 33%) or external compression (*n* = 8, 30%). Four patients received >1 aerstent TBY implantation and the total number of post‐interventional bronchoscopies ranged from 1 to 47, with mostly ≤5 flexible control bronchoscopies (59%) (Table 1).

In 11/27 (40.7%) patients, the initial stent was explanted after an average of 67.2 (±69.6) days. Interestingly, the stenting period in lung cancer patients was significantly longer (i.e., mean 131.8 [±80.1] days) than in esophageal cancer (i.e., mean 19.0 [±15.5] days), while stent explantation due to other causes was on average performed at day 44.4 (± 42.6) after implantation (one way ANOVA *p* = 0.047). Distribution of explanted and permanently remaining stents between palliatively and curatively intended stenting did not significantly differ (Fisher's exact test *p* = 0.130, data not shown) and average stenting period was insignificantly shorter in the curatively intended method (i.e., mean 39.6 [±43.2] days) than in palliatively assigned stenting (i.e., mean 105.8 [±85.4] days) (student's *t*‐test *p* = 0.167).

### Periprocedural and post‐interventional challenges

With regard to 31 aerstent TBY Y‐carina nitinol stent implantations, the median stenting period was 54 (IQR 19–160) days (Table 2). Intraprocedural complications included six at least partial airway obstructions of the right upper lobe bronchus and an incomplete unfolding of the stent in *two* cases. With regard to periprocedural complications, bleeding was solely apparent in endoluminal obstruction, but not in external compression or fistula (χ^2^ *p* = 0.034). Right upper lobe stent‐induced airway obstruction was comparable between indications: fistula (*n* = 1/10), external stenosis (*n* = 3/8) and endoluminal obstruction (*n* = 2/9) (χ^2^ *p* = 0.378). Yet, incomplete unfolding of the stent was only seen in patients with a tracheoesophageal fistula (*n* = 2/10), but not in external or endoluminal obstruction (χ^2^ *p* = 0.159). Cryotherapy, Nd:YAG laser therapy and argon plasma coagulation were foremost needed in cases of endoluminal obstructions (data not shown).

**TABLE 2:** Clinical characteristics, complications, and procedural outcome

	*n* of total procedures (31)	% of total (100.0)
Implantation complications		
Airway obstruction (right upper lobe bronchus)	6	19.4
Incomplete unfolding	2	6.5
Post‐implantation complications		
Mucus retention	25	80.6
Granulation tissue	19	61.3
Minor bleeding	6	19.4
Material defect	2	6.5
Dislocation	1	3.2
Migration (esophageal)	1	3.2
Explantation cause (*n* = 13/31)		
Granulation tissue	6	19.4
Dislocation	1	3.2
Tissue reconstitution	2	6.5
Material defect	1	3.2
Migration (esophageal)	1	3.2
Operative treatment/tracheostomy	2	6.5
Discharge/outcome		
Home/outpatient clinic	21	67.7
Rehabilitation clinic	1	3.2
Palliative care unit	2	6.5
In hospital death	7	22.6
Stenting period (days)		
0–30 days	11	35.5
31–60 days	7	22.6
61–90 days	1	3.2
91–120 days	1	3.2
121–150 days	3	9.7
151–180 days	3	9.7
181–210 days	4	12.9
> 210 days (i.e., 754 days)	1	3.2
Stenting period (days)		
Median (IQR)	54.0 (19.0–160.0)	
Time to discharge/stent explantation/death^a^ (days)		
Mean (± SD)	16.0 (± 17.1)	
Median (IQR)	10.0 (5.0–19.0)	

Abbreviations: SD, standard deviation; IQR, interquartile range.

a Depending on first chronological appearance.

tPost‐implantation complications were as a result of mucus retention (81%), granulation tissue (61%) and minor bleeding (19%). More relevant post interventional complications, such as material defect (*n* = 2), stent dislocation (*n* = 1) and tracheal perforation (*n* = 1) were rare, but often led to explantation. Other causes of stent explantation included formation of granulation tissue (*n* = 6), or an operative approach to airway management or underlying disease (*n* = 2) (Table 2). Average time from implantation to either discharge, stent explantation or death was 16.0 (± 17.1) days. After 21 (68%) interventions, the general condition of patients permitted their discharge back home with outpatient clinic control and one patient was transferred to a rehabilitation clinic. Two patients were transferred to a palliative care unit, while seven patients died in hospital.

### Survival analysis and outcome

**FIGURE 1:**
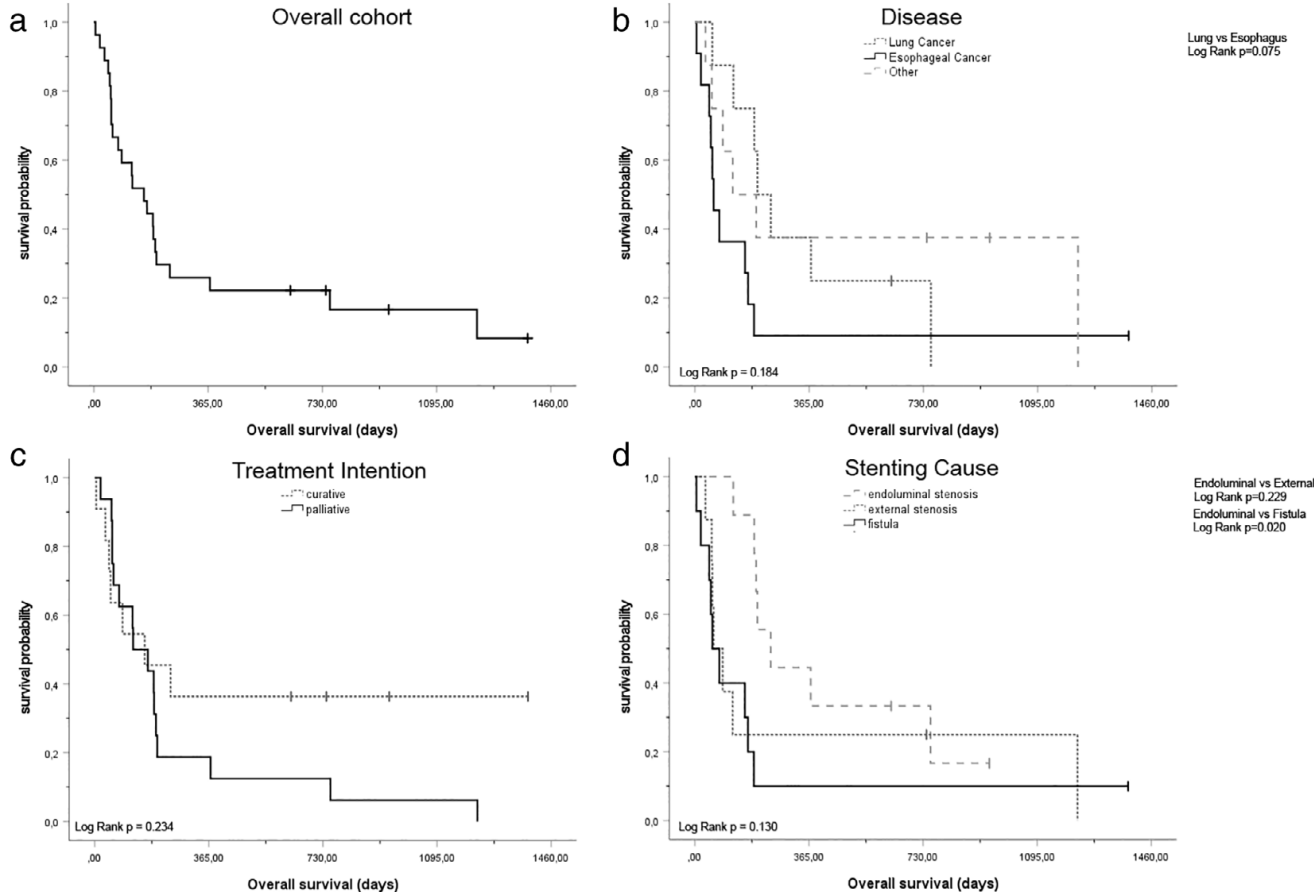
Survival analysis of the cohort. (a) Overall survival of the cohort (median survival [95% CI] 160 [76.9–243.1] days). (b) Overall survival with regard to underlying disease (lung cancer median survival [95% CI] 200 [126.5–273.5] days vs. esophageal cancer median survival [95% CI] 60 [30.9–89.1] days vs. other diagnosis median survival [95% CI] 121 [0.0–269.3] days). (c) Overall survival with regard on treatment intention (curative intention median survival [95% CI] 160 [0,0–367.2] days vs. palliative intention 49 [27.0–219.0] days). (d) Overall survival with regard on stenting cause (endoluminal stenosis median survival [95% CI] 243 [117.4–368.6] days vs. external stenosis median survival [95% CI] 60 [14.3–105.7] days vs. fistula median survival [95% CI] 56 [14.2–97.8] days)

Central airway stenosis is often an acute or subacute life‐threatening disease. Here, the presented cohort revealed an overall survival of 160 (95% CI: 76.9–243.1) days (Figure 1(a)). Nevertheless, survival of the underlying diseases varied heavily. While median overall survival in esophageal cancer was 60 (95% CI: 30.9–89.1) days, lung cancer survival leveled to 200 (95% CI: 126.5–273.5) days (log rank *p* = 0.075, Figure 1(b)). Other than that, pulmonary metastatic disease resulted in a median overall survival of 196 (95% CI: 0.0–423.2) days, but nonmalignant airway stenosis survival was as low as 121 (95% CI: 52.3–189.7) days. With respect to proximate implantation cause, tracheoesophageal fistula (median overall survival 56 [95% CI: 14.2–97.8] days) and external compression (median overall survival 60 [95% CI: 14.3–105.7] days) were low, but endobronchial obstruction revealed a median overall survival of 243 (95% CI: 117.4–368.6) days after stent implantation. Hence, stenting due to endobronchial obstruction showed a significant survival benefit in comparison with stenting due to tracheoesophageal fistula (log rank *p* = 0.02, Figure 1(d)) but not with stenting caused by external compression (log rank *p* = 0.229).

Interestingly, comparison of curatively and palliatively implanted stents did not result in a significant difference in overall survival (i.e., palliative situation median overall survival 123 [95% CI: 27.0–219] days vs. curative situation median overall survival 160 [95% CI: 0.0–367.2] days, log rank *p* = 0.234). During the first 6 months, Kaplan–Meier survival curves were completely in line (Figure 1(c)). Moreover, outcome was not altered by the urgency of stent implantation. An implantation due to an acute life‐threatening event (median overall survival 121 [95% CI: 38.9–203.1] days) resulted in comparable outcome with a subacute and well‐planned performance (median overall survival 160 [95% CI: 57.0–263.0] days, log rank *p* = 0.806).

### Case presentation

#### Case 1: Non‐small cell lung cancer in a 47‐year‐old female patient

**FIGURE 2:**
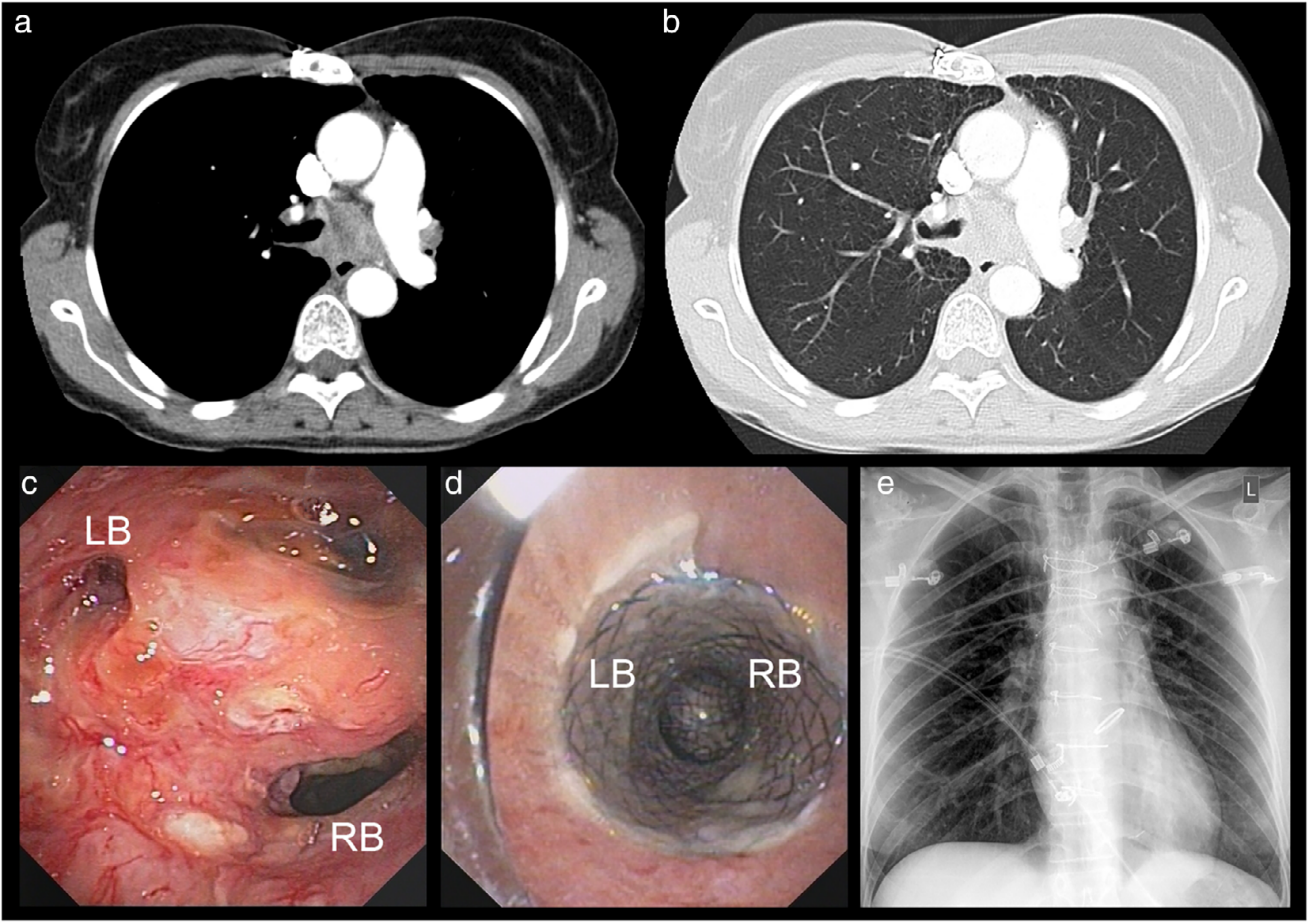
Case 1. (a) Contrast enhanced CAT‐scan in bone window setting, depicting mediastinal mass and occlusion of the left main bronchus. (b) Associated contrast enhanced CAT‐scan in lung window setting. (c) Videobronchoscopic view of the main carina, RB, right main bronchus; LB, left main bronchus. (d) Postinterventional videobronchoscopic view from above the stent with separation into the right main bronchus (RB) and left main bronchus (LB). (e) Post interventional chest X‐ray

A 47‐year‐old smoking female patient was transferred to the University Hospital Münster with hemoptysis under effective anticoagulation due to a mechanic valve in aortic position. The patient presented in a heavily reduced general condition (ECOG IV) with dyspnea. Previous diagnoses included Hodgkin's lymphoma, Ann Arbor stage IIA, curatively treated by 30 Gy mediastinal radiation at the age of 22 and coronary heart disease treated by bypass‐graft. Contrast‐enhanced CAT‐scan detected a mediastinal mass (Figure 2(a) and (b)). Emergency flexible bronchoscopy indicated central exophytic tumor growth with an almost completely occluded left main bronchus (Figure 2(c)). Forceps biopsy and further staging diagnostics resulted in a diagnosis of cT4 cN3 cM1, stage IVB non‐small cell lung cancer. After initial flexible bronchoscopy, rigid bronchoscopy was performed on the day of admission to implant a Y‐carina nitinol stent (30 mm tracheal, 15 mm right bronchus, 15 mm left bronchus) (Figure 2(d)). Chest X‐ray revealed correct positioning (Figure 2(e)). Five days after implantation, the patient was discharged and consecutively treated with cisplatin/vinorelbine plus 45 Gy mediastinal radiation therapy at an outpatient clinic. However, six months after initial stenting, the patient was readmitted due to progressive dyspnea resulting from a post stenotic pneumonia. After argon‐plasma‐coagulation (APC), the stent was successfully extracted, and the patient recovered on antibiotic treatment. While the patient was discharged without a tracheal stent, a stent reimplantation was needed 43 days after a discharge due to dyspnea caused by mediastinal tumor progression with left lung atelectasis. After left main bronchus recanalization by APC via rigid bronchoscopy, an aerstent TBY (40 mm tracheal, 20 mm right bronchus, 30 mm left bronchus) was placed. Without further relevant complications, the patient died 371 days after initial stent placement and 122 days after aerstent implantation as a result of underlying disease.

#### Case 2: Weaning failure after vascular prothesis implantation due to thoracic aortic aneurysm in a 25‐year‐old female patient

**FIGURE 3:**
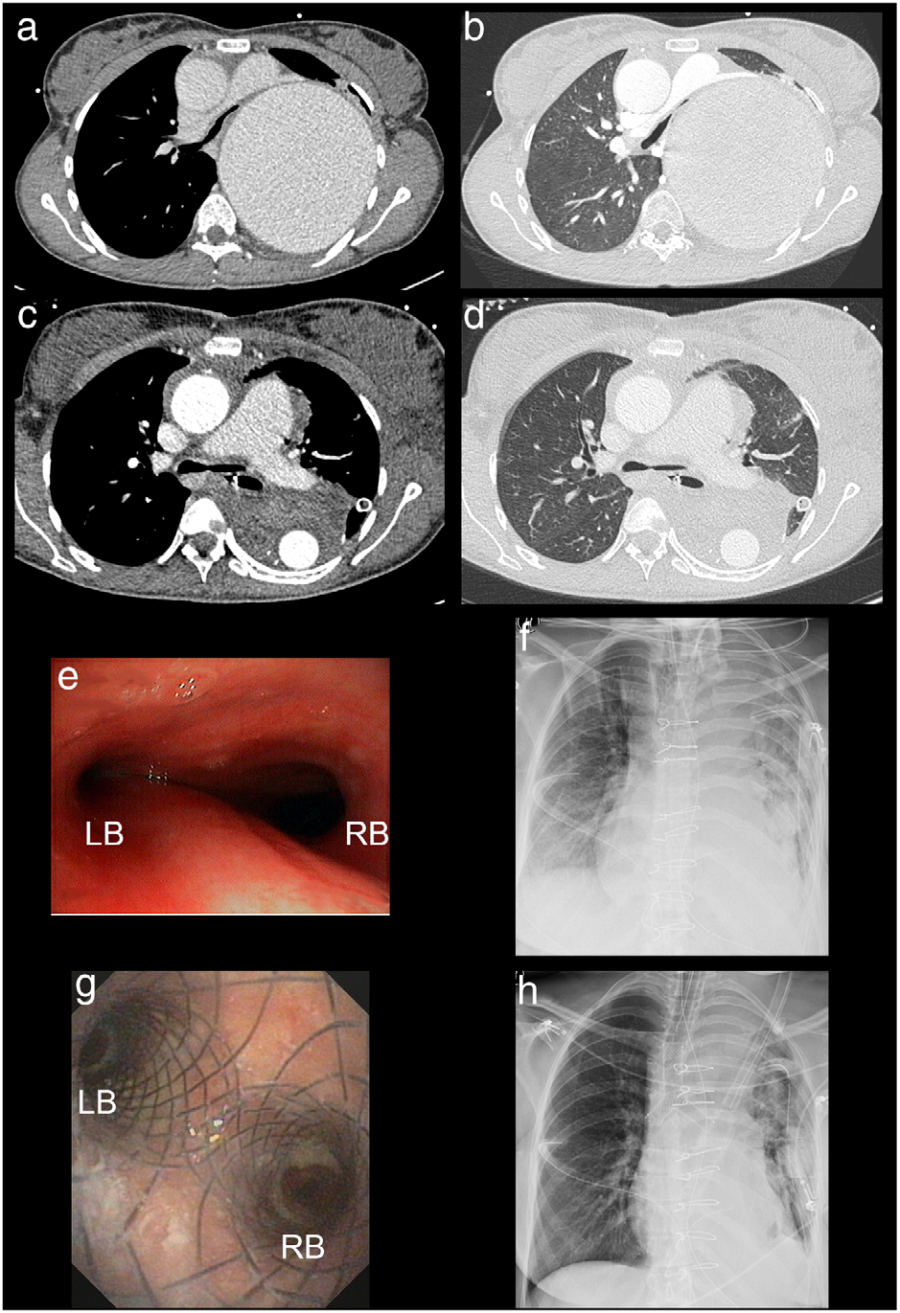
Case 2. (a) Late phase contrast enhanced CAT‐scan in bone window setting, depicting the thoracic aortal aneurysm with compression of the left main bronchus. (b) Associated contrast enhanced CAT‐scan in lung window setting. (c) Late phase contrast enhanced CAT‐scan in bone window setting, depicting the enhancement in the prosthetic conduit. (d) Corresponding contrast enhanced CAT‐scan in lung window setting with compression atelectasis of the left lower lobe. (e) Videobronchoscopic view of the main carina, RB marks right main bronchus, LB marks left main bronchus. (f) Preinterventional chest X‐ray with insufficient ventilation of the left lung. (g) Postinterventional videobronchoscopic of the main carina with separation into right main bronchus (RB) and left main bronchus (LB). (h) Postinterventional chest X‐ray with improved ventilation of the left lung

A 25‐year‐old female patient presented with a dry cough and progressive dyspnea at the University Hospital Münster. Apart from a treated arterial hypertension, no previous diagnoses were known, and the patient was in good condition. A contrast‐enhanced CAT scan revealed a 110 × 120 mm size aneurysm of the thoracic descending aorta (Figure 3(a),(b)), which was surgically treated with a prosthetic conduit (Figure 3(c),(d)). However, postoperative weaning failed due to left‐sided compression atelectasis as a result of a postoperative mediastinal hematoma (Figure 3(f)). Flexible bronchoscopy confirmed external compression of the left main bronchus (Figure 3(e)). Hence, indication was set for Y‐carina stent implantation. An aerstent TBY (40 mm tracheal, 20 mm right bronchus, 30 left bronchus) was implanted via rigid bronchoscopy (Figure 3(f)). Left lung ventilation subsequently improved (Figure 3(g)). Afterwards a successful weaning process allowed a transfer to standard care within 4 days. Discharge followed 15 days after stent implantation. However, 40 days after stent implantation, the patient was admitted to the emergency unit with dyspnea and fever. A left‐sided pneumonia was diagnosed due to retention of mucus at granulation tissue at the left stent end. Consequently, after antibiotic treatment, a stent extraction was performed with preceding APC treatment. Following this episode, no further airway stenting was needed.

#### Case 3: Esophageal carcinoma with esophageal‐tracheal fistula in a 55‐year‐old patient

**FIGURE 4:**
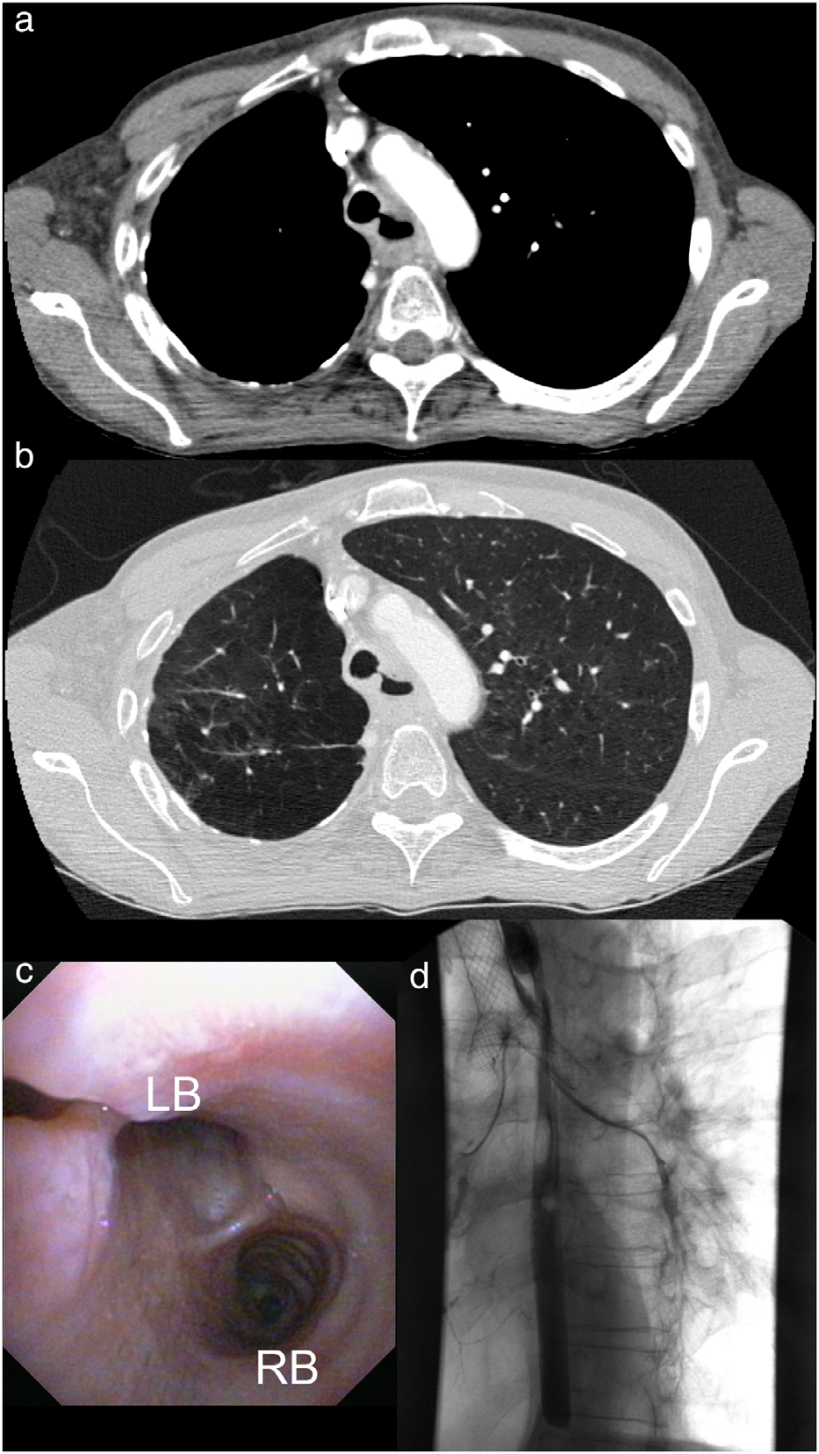
Case 3. (a) Contrast enhanced CAT‐scan in bone window setting, depicting tracheal esophageal fistula derived from the esophageal tumorous mass. (b) Associated contrast‐enhanced CAT‐scan in lung window setting. (c) Preinterventional videobronchoscopy with a view into the main carina. LB, left main bronchus; RB, right main bronchus. A tracheal eosphageal fistula is visible at the left upper corner of the image. (d) Postinterventional esophageal barium swallow resulted in bronchial contrast enhancement and correct stent positioning

A 55‐year‐old patient was admitted to the University Hospital Münster with a newly diagnosed esophageal‐tracheal fistula. The patient was incapable of oral intake due to recurrent aspiration and was in a heavily reduced general condition (ECOG III–IV). Three months earlier, the patient had been diagnosed with a stenosing squamous cell carcinoma of the esophagus (cT4a cNx cM0, stage IVA). Since then, the patient had been treated with definitive radiochemotherapy consisting of cisplatin and 5‐fluorouracil. A contrast‐enhanced CAT scan detected an esophageal‐tracheal fistula discharging into the left tracheal sulcus (Figure FIGURE 4(a),(b)). Flexible bronchoscopy proved direct esophageal penetration (Figure FIGURE 4(c)). An impassable esophageal stenosis at around 250–270 mm from the dental arch impeded esophageal stent implantation. Therefore, indication for tracheal stenting was set. In a rigid bronchoscopy, an aerstent TBY (40 mm tracheal, 20 mm right bronchus, 30 mm left bronchus) was implanted uneventfully. However, esophageal barium swallow resulted in bronchial contrast enhancement (Figure FIGURE 4(d)). After implantation of the bronchial stent, a sequential esophageal stenosis bougienage and implantation of a stent (Boston Scientific Wallstent 100 mm) was possible. Nevertheless, complications due to stent‐on‐stent situation had to be considered. Hence, a percutaneous endoscopic gastrostomy was later established. For the next few months, the patient was able to consume liquid orally and did not suffer aspiration pneumonitis. Alas, 3 months after tracheobronchial stenting, the patient presented with dysphagia, cough, and signs of aspiration. Flexible bronchoscopy revealed a fistula at about 2 cm above the proximal ending of the aerstent TBY with a visible penetration of the esophageal Wallstent into the tracheobronchial system. An additional tracheal stent (aerstent TBS, 60 mm tracheal) was successfully implanted without complications via rigid bronchoscopy. The patient died 189 days after initial Y‐carina nitinol stenting and 75 days after revisional tracheal stenting at home due to progressive esophageal carcinoma disease.

## DISCUSSION

Covered metal stenting is the most frequently used tracheobronchial stenting method, worldwide.^13^ Here, we add relevant data on the successful application of 31 self‐expanding, covered Y‐carina nitinol airway stents (Leufen aerstent TBY) in patients with central airway obstruction (CAO). Due to CAO, indication of airway stenting is often acute and life‐threatening. Most importantly, the short‐term survival of patients with palliative or curative treated underlying diseases did not alter (Figure 1(c)), and indication for central airway stenting should not primarily be influenced by the extent and treatment goal of an underlying disease. Hence, national guidelines even recommend the implementation of tracheobronchial stents in malignant CAO.^7^

In total, median overall survival (OS) of 160 days was comparable with other studies, that included patients treated with Y‐carina nitinol stents over the same period. While Gompelmann et al. presented a median OS of 122 days,^9^ Qiao et al. reported a median survival of 165 days,^2^ both with reduced frequency of esophageal carcinoma. In comparison with Madan et al., harboring a prominent proportion of patients with esophageal carcinoma (65.8%), 3 month mortality was at 47.4%,^3^ and at 40.7% in our cohort (data not shown). Taken together, survival is comparable with respect to the underlying disease. Of relevance, survival in our cohort was most promising in patients with endoluminal airway obstruction. Here, overall survival was significantly superior to patients with fistula but not to patients with external compression (Figure 1(d)).

Regarding complications, the major issues with covered and uncovered metal stents were granulation tissue formation and neoepithelialization^17^ in 15%–27% of the patients.^14^ Other than that, infection and hemoptysis have previously been reported to occur in up to 10% of cases.^14^

While Gompelmann et al. presented data on granulation tissue in 6/29 (20.7%) patients^9^ and Madan et al. described granulation tissue in 8/38 (21.1%) patients,^3^ in our cohort in 6/31 (19.4%) procedures, granulation tissue required explantation or stent exchange (Table 2). Nonetheless, overall presence of such was as high as 19/31 cases (61.3%). To confirm, each patient received at least a single planned follow‐up flexible bronchoscopy and we collected data on appearance of minimal to relevant granulation tissue. Hence, our data are more in line with the study of Ortiz‐Comino et al., which revealed granulation tissue in 19/46 Leufen aerstent cases (41.3%),^18^ yet only 10 of the 46 stents were Y‐carina nitinol stents.

Likewise, mucus plugging was documented in 84.8% of the aerstent cohort patients of Ortiz‐Comino et al.,^18^ which, despite frequent saline and bronchodilation inhalation, exactly reflects our incidence of 80.6%. However, the indication for planned follow‐up bronchoscopy has been widely discussed, as clinical symptoms and bronchoscopic evaluation do not necessarily match,^10^ possibly allowing for a symptom‐based approach.

However, the relevant complication of stent migration in our cohort was less frequent (Table 2) than in the aerstent cohort of Ortiz‐Comino et al. (13.0%).^18^ Here, data from the literature uncovered migration rates in malignant stenosis at 7.6% but at 32.5% in benign stenosis.^14^ Nonetheless, stent migration in benign stenosis in our cohort was not documented. Benign causes of central airway obstruction^11^ often request a short term intervention in an intensive care setting to promote weaning and extubation.^19^ Here, neoepithelialization and granulation tissue might have complicated the preplanned explantation procedure.

With regard to benign stenosis, we found a high proportion of patients with treated thoracic aortic aneurysms with mediastinal hematoma (*n* = 4/27). This has previously been rarely described, for example, by Reed et al.^20^ However, especially for benign stenosis, silicone Y‐stents are thought to be a superior method, as migration risk is inferior (i.e., 10% in malignant stenosis, and 18% in benign stenosis) compared to self‐expanding metal Y‐stents and symptomatic granulation tissue is observed in as few as 1% of the cases.^14^ In our cohort of self‐expanding Y‐shaped nitinol stents, granulation tissue was found in 3/5 patients and neoepithelialization was found in 2/5 patients treated for benign causes of CAO.

From our point of view, the inferior rigidness of the main bronchial stent sections in silicone Y‐shaped stents must be carefully considered against risks and complications during explantation of metal stents in cases of benign stenosis.^10^ Lachkar et al. recently published a comparative analysis of silicone Y‐shaped stents and self‐expanding metallic Y‐shaped stents, presenting data on significantly higher rates of stent failure in silicone stents (i.e., 7/40 procedures) than in self‐expanding metal stents (i.e., 0/38 procedures).^21^ This finding is completely in line with our clinical experience and unpublished data, as the opening force especially in developed stenosis or relevant external compression seems inferior in some silicone stent models compared to metal stents. Yet, a systematic review of Y‐shaped silicone stent placement in malignant (*n* = 285) and benign stenosis (*n* = 53) resulted in 98.3% successful deployments,^22^ now promoting intensified research efforts.

Overall, stent explantation in our cohort was performed in 40.7% of patients, which is higher than that presented in the study by Gompelmann et al. (i.e., 6.9% of the patients),^9^ or Lachkar et al. in the self‐expanding Y‐shaped metal cohort (i.e., 18.4% of the patients), as well as in the silicone Y‐shaped cohort (i.e., 26.5% of the patients).^21^ While the average stenting period was 54 days and rose to 67.2 days in cases of possible stent extraction, Lachkar et al. reported that it was 78 days in the self‐expanding metal stent cohort.^21^ Hence, data promote the feasibility of application of self‐expanding Y‐shaped nitinol stents, even for a limited period.

With respect to survival, the benefit in benign stenosis has not previously been documented for bronchial and tracheal stenting.^11^ In our cohort, the survival of patients with benign stenosis was comparably low (i.e., for benign stenosis median OS 121 [95% CI: 52.3–189.7] days vs. for malignant stenosis median OS 160 [95% CI: 32.4–287.6] days, log rank *p* = 0.808, data not shown). Yet, the need for endobronchial stenting in massive aortic aneurysm is rare and prospective trials are therefore not warranted. Hence, interdisciplinary discussion regarding stenting should always be discussed for these critically ill patients.

In conclusion, application of self‐expanding, covered Y‐carina nitinol metal stents (Leufen aerstent TBY) is a feasible and safe procedure to retain bronchial patency. Underlying diagnoses should not impede implantation, but periprocedural as well as post interventional complications must be weighed against direct benefits and should direct the choice of stenting material. Stenting almost directly improves ventilation,^19^ and hence might be a conceivable treatment option, even in morbid patients to reduce anxiety, stress and improve performance^5^ and quality of life.^1^, ^11^

## CONFLICT OF INTEREST

None of the authors report any conflict of interest.
